# An Intrinsic Propensity of Murine Peritoneal B1b Cells to Switch to IgA in Presence of TGF-β and Retinoic Acid

**DOI:** 10.1371/journal.pone.0082121

**Published:** 2013-12-06

**Authors:** Bishnudeo Roy, Anne-Margarete Brennecke, Shiwani Agarwal, Martina Krey, Sandra Düber, Siegfried Weiss

**Affiliations:** Molecular Immunology, Helmholtz Centre for Infection Research, Braunschweig, Lower Saxony, Germany; DRFZ, Germany

## Abstract

**Aims:**

In the present study we have investigated the comparative switching propensity of murine peritoneal and splenic B cell subpopulations to IgA in presence of retinoic acid (RA) and TGF-β.

**Methods and Results:**

To study the influence of RA and TGF-β on switching of B cell subpopulations to IgA, peritoneal (B1a, B1b and B2 cells) and splenic (B1a, marginal zone, and B2) B cells from normal BALB/c mice were FACS purified, cultured for 4 days in presence of RA and TGF-β and the number of IgA producing cells was determined by ELISPOT assay or FACS analysis. In presence of TGF-β, peritoneal B1b cells switched to IgA more potently than other peritoneal B cell subpopulations. When TGF-β was combined with retinoic acid (RA), switching to IgA was even more pronounced. Under these conditions, “innate” B cells like peritoneal and splenic B1 cells and MZ B cells produced IgA more readily than B2 cells. Additionally, high frequency of nucleotide exchanges indicating somatic hypermutation in VH regions was observed. Besides IgA induction, RA treatment of sorted PEC and splenic B cells led to expression of gut homing molecules - α_4_β_7_ and CCR9. Intraperitoneal transfer of RA-treated B1 cells into Rag1^-/-^ recipients resulted in IgA in serum and gut lavage, most efficiently amongst B1b cell recipients.

**Conclusion:**

Present study demonstrates the differential and synergistic effect of RA and TGF-β on switching of different B cell subpopulations to IgA and establishes the prominence of peritoneal B1b cells in switching to IgA under the influence of these two factors. Our study extends our knowledge about the existing differences among B cell subpopulations with regards to IgA production and indicates towards their differential contribution to gut associated humoral immunity.

## Introduction

IgA is the most abundant class of antibodies present in mammalian mucosal tissues. It forms a first-line of defense against invasion by inhaled or ingested pathogens and plays an important role in the maintenance of immune homeostasis. Besides mucosal tissues, IgA is also found at significant concentrations in the serum of many species, where it mediates the elimination of pathogens that have breached the mucosa.[1]

Class switch recombination (CSR) to IgA is orchestrated by various cytokines and other factors.[2]–[4] Amongst them, TGF-β and retinoic acid (RA) are most prominent.[2], [5] A special role of TGF-β in IgA CSR is most evident from the observation that mice deficient for TGF-β or lacking TGF-β receptor II expression on B cells exhibit reduced levels of IgA.[6], [7] In gut, TGF-β is produced by B cells (autocrine factor) [8], [9], T cells [10] dendritic cells (DCs) [11], and stromal cells.[12] Some of the T cells that produce TGF-β are claimed to be Foxp3^+^ CD4^+^ regulatory T cells.[2] Besides TGF-β, vitamin A metabolite RA is also a highly potent inducer of IgA CSR.[5] RA is produced by gut associated DCs and macrophages.[13]–[15] In accordance, the generation of IgA secreting cells (SCs) and their homing to gut is promoted by intestinal DCs and appears to be dependent on RA.[13] Consistently, mice deficient in RA precursor vitamin A showed reduced numbers of IgA producing cells in the small intestine even though the IgA levels in the serum remained unchanged.[13] The interplay between TGF-β and RA is still controversially discussed. It has been demonstrated that TGF-β inhibits RA induced IgA CSR.[13] However, another study using splenic cells showed that a combination of RA and TGF-β with additional factors (LPS, APRIL, and IL5) acts synergistically to induce IgA switching *in vitro*.[16]

In addition to TGF-β and RA, cytokines, like IL2, IL4, IL5, IL6 and IL10, are also thought to induce CSR and promote IgA production either via TGF-β induction within B cells or by enhancing the post-switch maturation.[4] Furthermore, cytokines of the tumor necrosis factor (TNF) ligand family called B cell activating factor (BAFF/BLyS) and a proliferation inducing ligand (APRIL) produced by gut DCs are also considered to be involved in IgA switching.[17], [18]

In mice, at least three mature B cell subpopulations – follicular (FO), marginal zone (MZ) and B1 B cells constitute the humoral immune response, where B1 cells can be subdivided further into B1a and B1b cells.[19], [20] FO B cells also known as B2 cells are present in the B cell follicles of secondary lymphoid organs and respond to antigens T cell dependently. On the other hand, MZ B cells residing in the splenic marginal zone and B1 cells predominantly located in the peritoneal cavity (PEC) or pleural cavity, are believed to respond to antigens without T cell help and play an important role in the early stages of pathogenic invasion.[21] Such B cells respond differentially to various stimuli and need multiple triggers to induce IgA class switching.[16], [22]–[24] Thus, under minimal stimulatory conditions (BLys+LPS+TGF-β) they switch to IgA preferentially in comparison to B2 cells from PEC or spleen.[24]

The contribution of B1 cell subsets to IgA production *in vivo* is not known. In this regard, we could recently show that most of the IgA producing cells in the PEC of unmanipulated mice belonged to B1b cell population.[25] However, differential switching of B1a and B1b cells to IgA under stimulatory culture conditions has not been comparatively studied. In addition, the combination of TGF-β and RA that was supposed to have synergistic effects on naïve splenic B cells to switch to IgA [16] was never tested on various peritoneal and splenic B1 cell subsets.

Thus, in the present work, we stimulated peritoneal B1a, B1b and B2 cells with LPS, BLys and TGF-β *in vitro*. Switching to IgA was severely enhanced in peritoneal B1b cells in comparison to their B1a and B2 counterparts. Switching to IgA was further enhanced when TGF-β was combined with RA and IL5. *In vitro* switched B1 cells also showed the presence of frequent nucleotide exchanges in their functionally rearranged VH gene segments. This indicates that besides IgA CSR, somatic hypermutation had also taken place. These findings could be extended in part also to splenic B1a and MZ B cells. Altogether, these findings demonstrate differential switching of B cell subpopulations to IgA in response to various stimuli *in vitro*. This suggests alternative mechanisms regulating the IgA CSR of different B cell subsets *in vivo*.

## Materials and Methods

### Ethic Statement

All experiments were performed in accordance with the German Law on Care and Use of Laboratory Animals and were approved by the local authority (LAVES) under permission number 33.9-42502-04-11/0390.

### Mice

Normal BALB/c mice, 7–10 weeks old, used for the experiments were purchased from Janvier. Rag1^-/-^ mice on BALB/c background were obtained from our animal facility (Helmholtz Institute for Infection Research; Braunschweig, Germany).

### Cell preparation, FACS analysis and cell sorting

To prepare PEC cells, peritoneum was flushed two times with 8 ml of normal cell culture medium (IMDM), collected washout was centrifuged and the pellet was resuspended in appropriate amount of cell culture medium. Monoclonal antibodies against mouse CD19, CD5, CD43, Mac-1, IgA, IgM, CD23, CD21, CCR9 and α_4_β_7_ conjugated with FITC, PE, APC, PE-Cy7, or Biotin were obtained from Pharmingen or eBioscience or were home made. Biotinylated antibodies were revealed by Streptavidin-PerCPCy5.5 (eBioscience). Flow cytometry was done using LSR II (BD) and data were analyzed using FACS DIVA software. Cell sorting was performed using FACSAria® (Becton Dickinson). Reanalysis showed that cells were >95% pure

### RT-PCR

Total RNA from sorted/cultured cells was prepared with TriFast™ FL Reagent (peQLab) following manufacturer's protocol. DNase (Qiagen) treated RNA was reverse transcribed using oligo-d(T)_12–18_ (Thermo Scientific) and RevertAid™ reverse transcriptase (Fermentas). PCR amplification of cDNA was performed using HotstarTaq™ DNA polymerase (Qiagen). and the following primers: Igµ/α heavy chain variable (VH) region, for VHcons 5′-GAGGTGCAGCTGCAGGAGTCTGG-3′ rev Cµ2 5′-CATTTGGGAAGGACTGA-3′ or Cα2 5′-GAGCTGGTGGGAGTGTCAGTG-3′.

### Sequencing of Ig VH chain and sequence analysis

RT-PCR products of IgVH chain transcripts from 4 days cultured cells were cloned using TOPO TA Cloning® kit (Invitrogen) following manufacturer's protocol. Plasmids were isolated using QIAprep Spin Mini Prep Kit (Qiagen). Sequencing was done using M13 Reverse primers by the department of Genome Analysis of Helmholtz Centre for Infection Research, Braunschweig. IgA and IgM VH sequences derived from sorted PEC and splenic B cells were submitted to GenBank (accession no. KF207924 – KF208284).

### Cell culture and ELISPOT assay

Purified peritoneal and splenic B cell populations, resuspended in IMDM at a density of approximately 1×10^5^ cells/ml were stimulated with, LPS (25 µg/ml; *Escherichia coli*, Sigma), Recombinant Mouse BLys (100 ng/ml; R&D SYSTEMS), Recombinant Human TGF-β1 (2 ng/ml; R&D SYSTEMS), IL4 (1∶400; home made), IL5 (1∶400; home made), and all trans-Retinoic Acid (100 nM; Sigma). ELISPOT: single-cell suspensions at serial dilutions of 1∶5 were plated on plates coated with anti-mouse IgA (Pharmingen) or anti-mouse IgM (Pharmingen). Cells were incubated overnight at 37°C with 5% CO_2_ and 95% humidity. After washing with PBS+0.01% Tween 20, biotinylated anti-mouse IgA (eBioscience) or anti-mouse IgM (SeroTec) was added. Bound antibodies were developed with Streptavidin-horseradish peroxidase using AEC (3-amino-9-ethyl-carbazole; Sigma) in DMF (N,N-Dimethylformamide; Sigma) diluted in 0.1 M acetate solution added with H_2_O_2_ as substrate. Spot development was stopped by washing plates with water and spots marking antibody secreting cells (ASCs) were counted after drying.

### ELISA

Ig concentrations in serum or intestinal lavage were measured by ELISA. In brief, 96 well plates (MaxiSorb TM Immunoplates, Nunc) were coated over night with anti-mouse IgA (Pharmingen) or anti-mouse IgM (Pharmingen) or goat-anti-mouse IgG (Sigma) antibodies at 4 C and blocked for 1 h with 3% BSA in 0.05% Tween 20. Appropriately diluted culture supernatant/sera/intestinal lavage from each mouse was added to the wells and incubated for 2 h at RT. Bound IgA/IgM/IgG was detected with biotinylated rat anti-mouse IgA (eBioscience) or rat-anti-mouse IgM (SeroTec) or PO conjugated goat-anti-mouse IgG (Jackson ImmunoResearch) antibodies. Biotinylated antibodies were revealed by using horseradish peroxidase (HRP) conjugated streptavidin (BD). Bound HRP activity was visualized using o-Phenylendiamin (OPD) as substrate and the results were read using an ELISA-reader (BioRad 3550- UV microplate reader).

### *In vivo* adoptive transfer

Sort purified PEC B1a and B1b cells cultured under different stimulatory conditions for 3 days were collected, washed, resuspended in PBS and injected i.p into Rag1^-/-^ hosts.

### Statistics

Paired two-tailed Student's *t*-test was applied to determine the statistical significance (*p* value). *p*≤0.05 was considered significant. *p<0.05; **p<0.01; ***p<0.001.

## Results

### Peritoneal cavity B1b cells switch preferentially to IgA in presence of TGF-β

**Figure 1:**
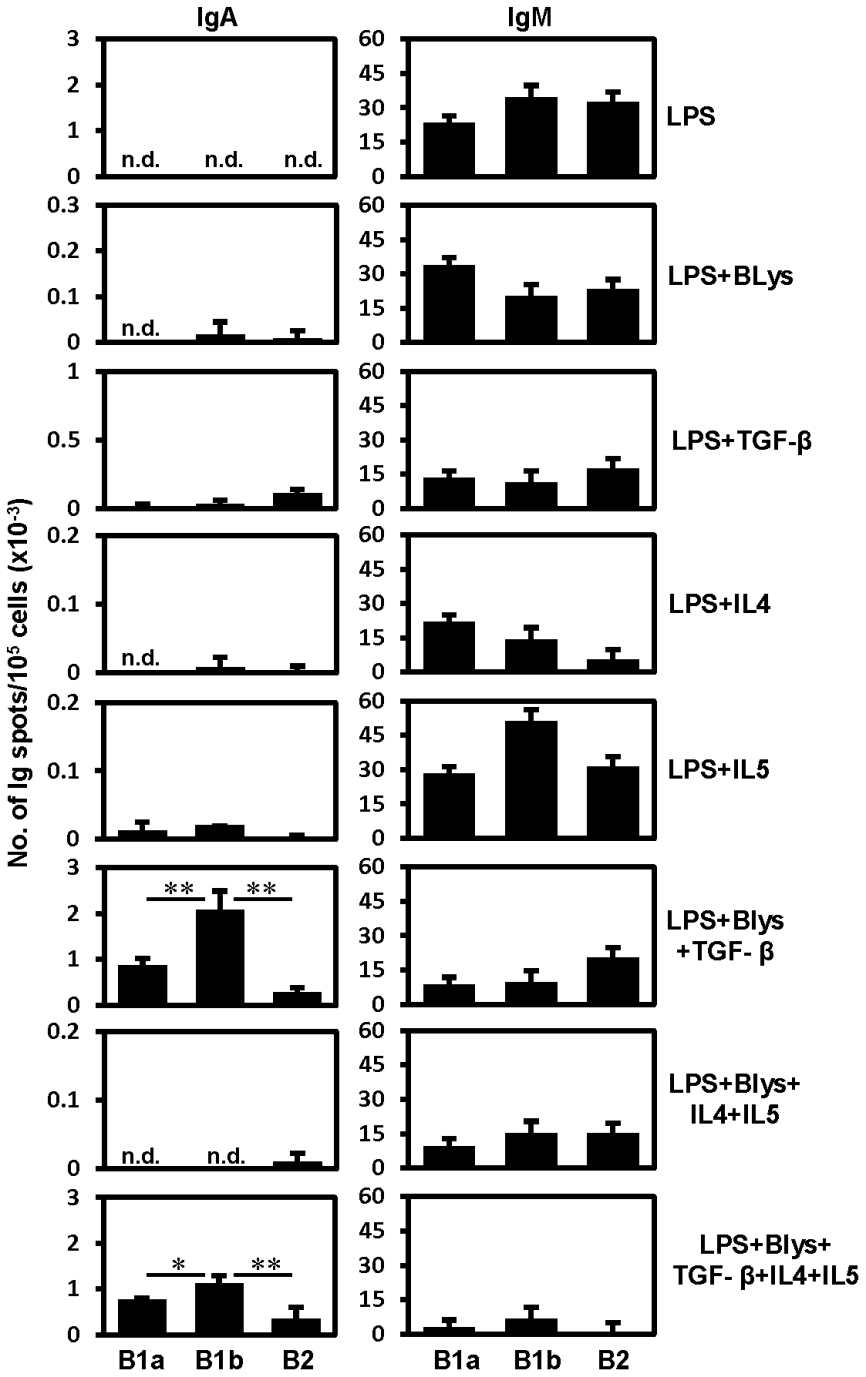
TGF-β enhances IgA switching among PEC B1b cells *in vitro*. Results of IgA and IgM ELISPOT assays performed in triplicates on sort purified PEC B cell subpopulations after 4 days of treatment with the indicated combination of stimulatory factors in culture. Surface IgA^+^ cells were excluded by sorting from B1a (IgA^-^CD19^hi^CD5^+^CD43^+^Mac-1^+^), B1b (IgA^-^CD19^hi^CD5^-^CD43^+^Mac-1^+^) and B2 (IgA^-^CD19^lo^CD5^-^CD43^-^Mac-1^-^) B cell subpopulations. Data show the results of one of three independent experiments. Bars represent mean ± SD.

TGF-β has been known to promote switching of B cells to IgA in general.[2], [26], [27] Under the influence of this cytokine, combined with LPS and Blys, differential propensity of switching to IgA was observed among PEC and splenic B cell subtypes.[24] Interestingly, under such conditions, PEC B1 and splenic MZ B cells switched *in vitro* to IgA more prominently in comparison to PEC or splenic B2 cells. However, in this study, B1 cells were not differentiated into B1a and B1b cells and thus their differential capability to switch to IgA was not established.[24] Our previous *in vivo* finding had suggested differences between PEC B1a and B1b cells in that respect.[25] This prompted us to test their preferences to switch to IgA under *in vitro* stimulatory conditions. For this purpose, PEC B cells were FACS purified into – B1a, B1b and B2 cells (Figure S1). B cells expressing surface IgA were sorted out to exclude expansion of such cells *in vitro*. After 4 days of stimulation in the presence of different combinations of cytokines, IgA and IgM ELISPOT assays were applied to assess the number of IgA and IgM SCs. Under different stimulatory conditions, the combinations of LPS+Blys+TGF-β and LPS+Blys+TGF-β+IL4+IL5 led to significant switching amongst cultured B cells (Figure 1). In comparison to B2 cells, both B1 cell subpopulations showed higher numbers of IgA SCs (Figure 1). Quite interestingly, B1b cells produced constantly higher number of IgA SCs in comparison to B1a cells (Figure 1). Presence of BLys was required for efficient IgA CSR as treatment with LPS+TGF-β alone led to generation of very few IgA SCs (Figure 1).

Parallel, ELISPOTs for IgM showed no apparent trend for the number of IgM SCs amongst three PEC B cell populations under different stimulatory conditions (Figure 1). However, a decrease in the number of IgM SCs was noted for conditions under which an increase in the numbers of IgA SCs had been observed (Figure 1).

### Pronounced switching of B1 cells to IgA when TGF-β is combined with RA

**Figure 2:**
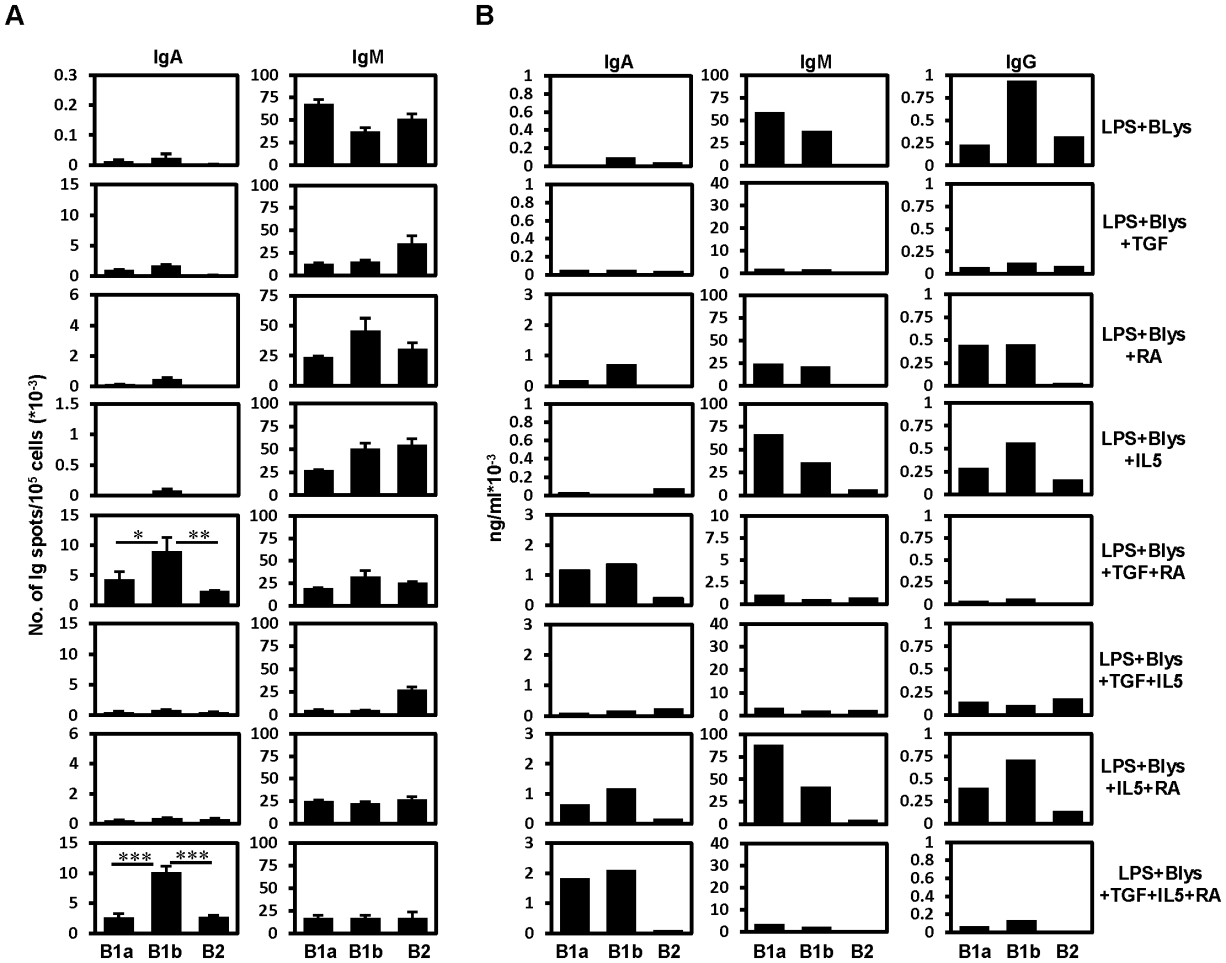
TGF-β in combination with RA synergistically induces IgA switching by PEC B cells. (A) Results of IgA and IgM ELISPOT assays performed in triplicates on sort purified PEC B cell subpopulations after 4 days of treatment with the indicated combination of stimulatory factors in culture. Surface IgA^+^ cells were excluded by sorting from B1a (IgA^-^CD19^hi^CD5^+^CD43^+^Mac-1^+^), B1b (IgA^-^CD19^hi^CD5^-^CD43^+^Mac-1^+^) and B2 (IgA^-^CD19^lo^CD5^-^CD43^-^Mac-1^-^) B cell subpopulations. (B) Amount of secretory Igs determined by ELISA in the supernatant of B cells cultured for 4 days under the indicated stimulatory conditions. Data show the results of one of two independent experiments. Bars represent mean ± SD.

RA has been shown to promote the switching of naïve splenic B cells to IgA.[16] The differential effect of this vitamin A metabolite on various PEC and splenic B cell subpopulations has not been studied so far. Therefore, to examine the influence of RA on PEC and splenic B cell subpopulations, sorted peritoneal and splenic B cells were subjected *in vitro* to different combinations of stimulatory factors including RA. After 4 days of activation, the number of IgA and IgM SCs were measured by ELISPOT assay. Consistent with earlier findings, considerably high numbers of IgA SCs were observed in the PEC B cell cultures supplemented with RA plus TGF-β, although these reagents alone in combination with other factors induced comparatively low numbers of IgA SCs (Figure 2A). Interestingly, under these circumstances, again PEC B1a and B2 cells had significantly less number of IgA ASCs than B1b cells. No differences in the numbers of IgM ASCs were observed under the condition (LPS+Blys+TGF-β+IL5+RA) in which, very high number of IgA ASCs was observed (Figure 2A). This finding was further confirmed by ELISA that was done to determine the amount of IgA in the culture supernatants of the cultured PEC B cells (Figure 2B). Consistently, PEC B1 cells also showed the expression of *Igα* germ line transcripts after combined treatment with TGF-β and RA (Figure S2). When tested for the reactivity against gut bacteria, secretory IgA present in the culture supernatant of PEC B cells showed their binding towards these commensals (Figure S3). This is consistent with our previous finding that showed the binding of B1 cell derived Igs to gut bacteria [28]. Furthermore, analysis of secretory IgG in the culture supernatants showed that the amount of IgG decreased to a minimum under conditions that favored the switching to IgA (Figure 2B). Interestingly, under other conditions, the amount of secretory IgG was found to be higher in B1b cell culture supernatants in comparison to the other two PEC B cell populations (Figure 2B).

**Figure 3:**
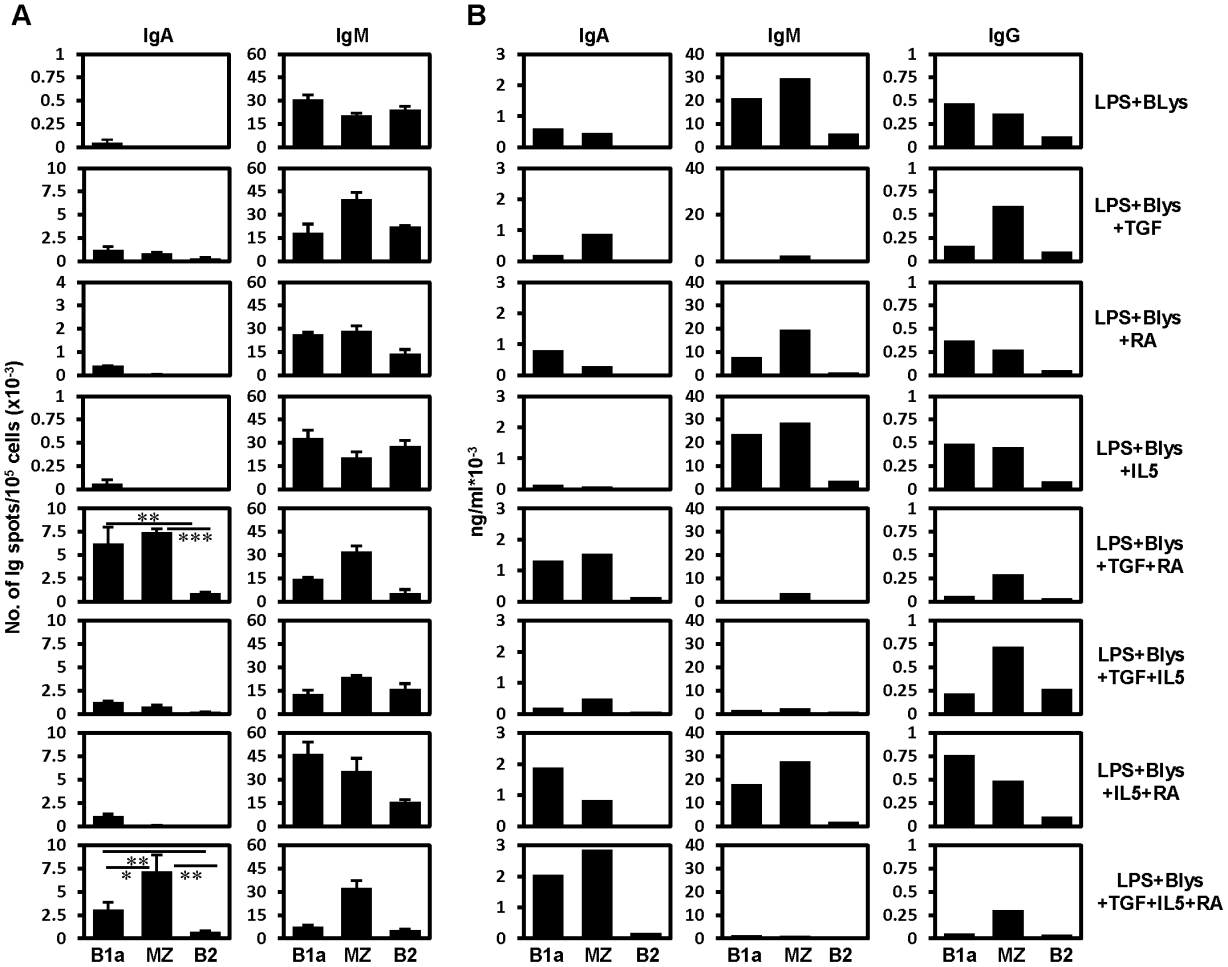
TGF-β in combination with RA synergistically induce IgA switching in splenic B1a and MZ B cells. (A) Results of IgA and IgM ELISPOT assays performed in triplicates on sort purified splenic B cell subpopulations after 4 days of treatment with the indicated combination of stimulatory factors in culture. Surface IgA^+^ cells were excluded by sorting from B1a (IgA^-^CD19^+^CD5^+^CD23^-^CD21^lo/-^), MZ (IgA^-^CD19^+^CD5^-^CD23^lo/-^CD21^hi^) and B2 (IgA^-^CD19^+^CD5^-^CD23^hi^CD21^int^) B cell subpopulations. (B) Amount of secretory Igs determined by ELISA in the supernatant of B cells cultured for 4 days under the indicated stimulatory conditions. Data show the results of one of two independent experiments. Bars represent mean ± SD.

Among splenic B cell subpopulations (FACS sorted according to the plan showed in Figure S1), mainly B1a and MZ B cells underwent pronounced switching to IgA compared to splenic B2 cells when treated with TGF-β plus RA (Figure 3A, B). Addition of IL5 to the combination of RA and TGF-β did not increase the number of IgA ASCs (Figure 3A). Presence of secretory IgA in the supernatants of cultured B cells as determined by ELISA was consistent with the ELISPOT results (Figure 3A, B). IgG production seemed to take place without the addition of TGF-β or retinoic acid. However, TGF-β or RA appeared respectively to suppress or enhance the production of IgG in case of splenic B1a cells, (Figure 3B).

Cell counts of cultured cells indicated strongly reduced proliferation of all PEC and splenic B cell types in presence of TGF-β and RA wherever IL5 was not added (Table 1). This is consistent with the recent finding showing strong inhibition of B cell proliferation in the cultures added with RA and TGF- β [29]. In addition, these B cell types showed an uneven increase in their number under various culture conditions (Table 1). This might account for the slight differences between ELISPOT and ELISA data, later being only qualitative under these circumstances.

**Table 1:** Increase in cell numbers after 4 days of culture under various conditions.

	Fold increase in no. of cells after 4 days of culture^1^					
	PEC			Spleen		
Culture condition	B1a	B1b	B2	B1a	MZ	B2
**LPS+BLys**	5.90±1.59	7.20±0.42	3.10±0.65	6.54±1.31	6.88±3.75	3.12±1.70
**LPS+Blys+TGF-β**	2.93±0.39	2.33±1.73	1.60±0.56	1.81±0.71	1.93±0.98	0.79±0.40
**LPS+Blys+RA**	6.23±1.59	3.55±0.62	1.55±0.70	2.94±2.03	5.07±2.56	0.96±0.42
**LPS+Blys+IL5**	12.84±5.79	8.34±1.54	3.67±1.64	4.88±2.62	6.91±3.25	2.16±0.84
**LPS+Blys+TGF-β+RA**	1.27±0.49	0.63±0.24	0.32±0.27	0.66±0.56	0.48±0.26	0.25±0.15
**LPS+Blys+TGF-β+IL5**	6.06±1.38	5.49±1.09	1.83±0.38	2.50±1.41	3.72±1.29	1.04±0.34
**LPS+Blys+RA+IL5**	15.84±5.53	16.54±5.92	2.44±0.18	4.45±0.98	4.94±1.06	0.99±0.55
**LPS+Blys+TGF-β+RA+IL5**	7.54±1.55	3.92±1.43	0.49±0.16	4.70±2.87	1.05±0.48	0.46±0.32

^1^ Sorted IgA^-^ PEC and splenic B cells were cultured under different conditions for 4 days and the fold increase in cell number was calculated by dividing the cell count after 4 days by the number of cells at the beginning of the cell culture. The values represent mean ± SD calculated from three independent culture experiments.

### *In vitro* differentiated B1 cells also exhibit somatic hypermutations

**Figure 4:**
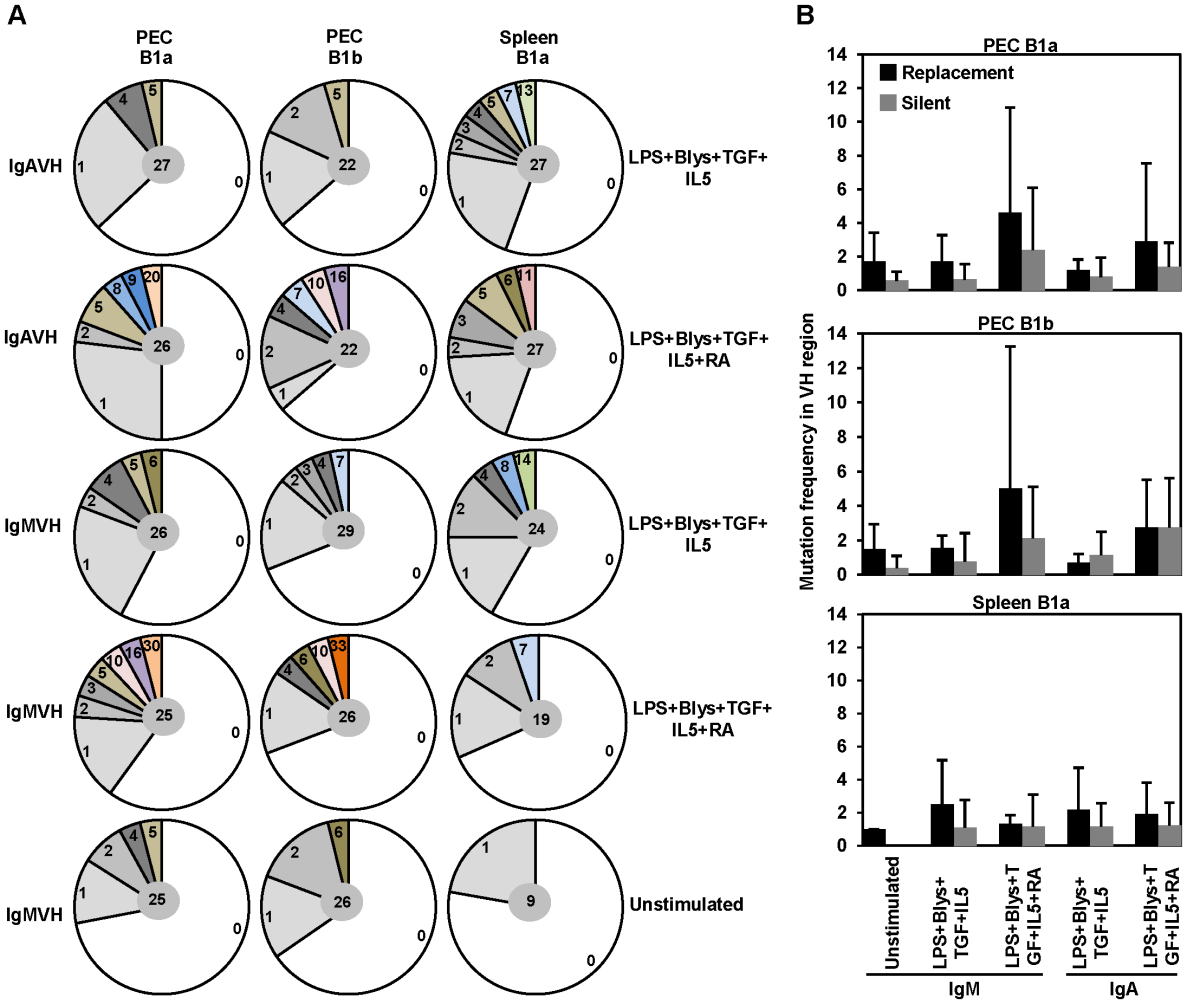
PEC and splenic B1 cell derived IgA VH sequences display high frequencies of nucleotide exchanges due to somatic hypermutation. Frequency of mutations observed in the IgM or IgA VH region sequences derived from PEC and splenic B1 cells cultured for 4 days with the indicated combinations of IgA CSR inducing factors. (A) Each colored sector represents a particular number of mutations found at a particular frequency. Total number of sequences is shown in the middle. (B) Number of replacement and silent mutations amongst IgM and IgA VH sequences derived from indicated B cell populations. Sequences were analyzed using SEQUENCHER™ Version 4.1 for Macintosh software (Gene Codes Corporation). Assignment of VH chain was done using VBASE2 database (http://www.vbase2.org/). Only functional sequences were included for the mutation analysis. Bars represent mean ± SD.

The enzyme AID responsible for CSR also mediates somatic hypermutation (SHM). Consistent with that, we had detected the expression of AID among B cell populations cultured under stimulatory conditions (data not shown). Thus, quite expectedly, analysis of IgA VH sequences from PEC B1 cell and splenic B1a cell cultures supplemented with TGF-β or a combination of TGF-β and RA exhibited the presence of frequent mutations (Figure 4A). Mutation frequencies observed under IgA CSR inducing culture conditions were higher compared to unstimulated B cells (Figure 4A). Interestingly, frequent nucleotide exchanges were also detected amongst IgM VH sequences derived from *in vitro* stimulated B1 cells, (Figure 4A). Almost all of the VH sequences were found to be functional and devoid of stop codons or frame shift mutations in the coding VH region (Table 2). Analysis of replacement (R) vs. silent (S) mutation revealed a high R/S ratio in the VH regions (Figure 4B).

**Table 2:** Nonfunctional^1^ vs functional sequences.

Sequence	Condition	PEC B1a	PEC B1b	Spleen B1a
**IgAVH**	LPS+Blys+TGF+IL5	1/27	1/22	1/27
**IgAVH**	LPS+Blys+TGF+IL5+RA	1/26	2/22	1/27
**IgMVH**	LPS+Blys+TGF+IL5	2/26	0/29	3/24
**IgMVH**	LPS+Blys+TGF+IL5+RA	1/25	3/26	0/19
**IgMVH**	Unstimulated	2/25	0/26	2/9

^1^ Sequences having frame shift or stop codon in the VH coding region were considered to be nonfunctional. The numbers of nonfunctional vs. functional sequences are displayed below indicated cell population.

### Retinoic acid enhances the expression of gut homing molecules by B1 cells

**Figure 5:**
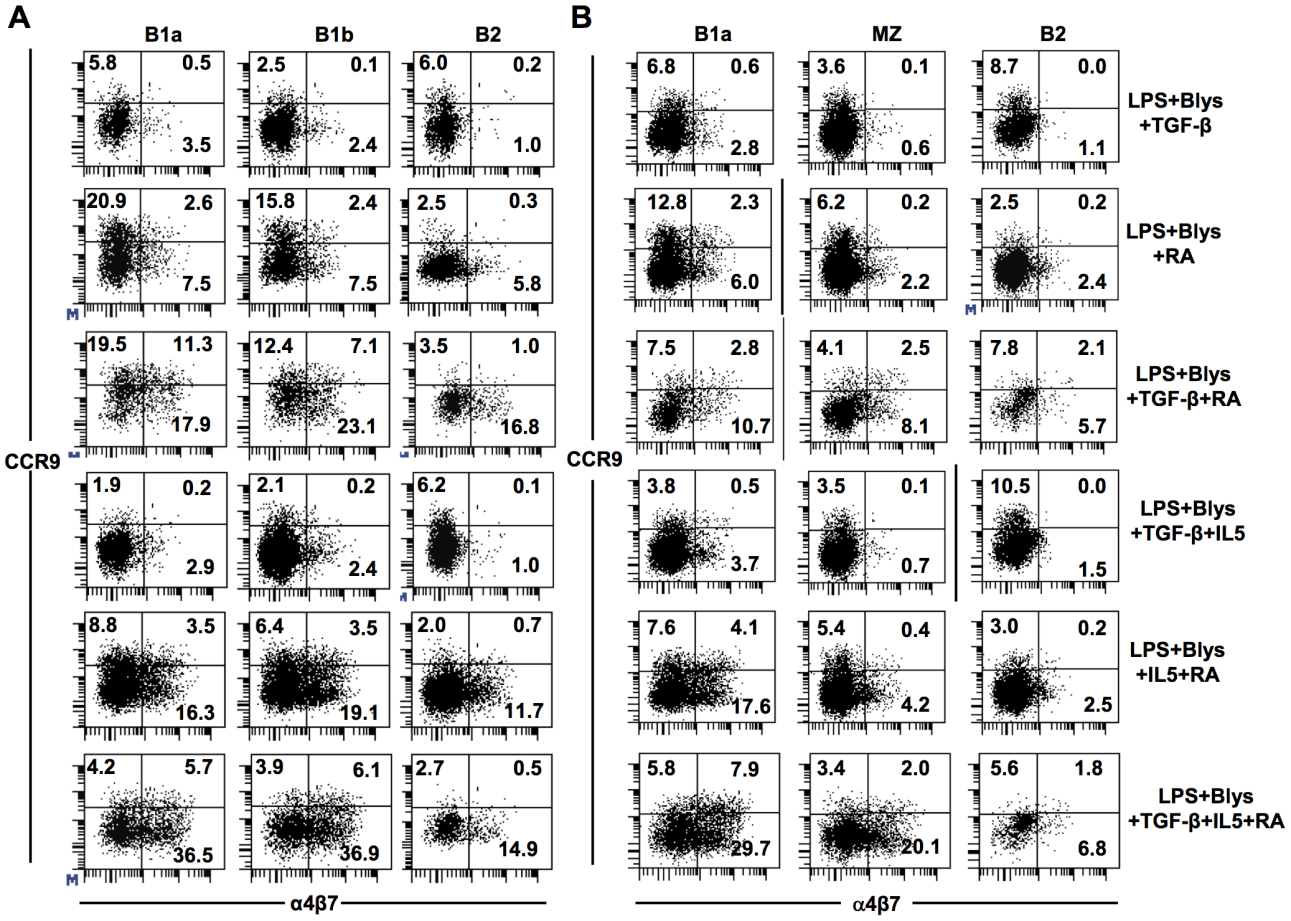
RA treatment leads to up-regulation of gut homing molecules on the surface of cultured PEC B cells. Expression of surface α4β7 and CCR9 on IgA^-^ PEC (A) or splenic (B) B cells sorted in fashion similar to previous experiments was checked by flow cytometry after four days of culture under various combinations of stimulatory conditions. Using the same cultures (as in A and B), expression of surface IgA and IgM on PEC (C) and splenic (D) B cells was checked by flow cytometry. Data show the results of one of two independent experiments.

Besides the induction of IgA switching, retinoic acid is known to induce the expression of gut homing surface receptors (α4β7 and CCR9) on target B and T cells.[5], [14] CCR9 is a chemokine receptor for CCL25 which is secreted by gut epithelial cells and α4β7 is an integrin that binds to the mucosal adressin MadCAM.[30], [31] Expression of these two molecules was expected to be induced after *in vitro* treatment of peritoneal and splenic B cell subpopulations with TGF-β and RA as IgA producing plasma blasts are supposed to home to intestine. Consistent with earlier findings, treatment with RA led to high surface expression of α4β7 and/or CCR9 on some of the treated PEC and splenic B cells (Figure 5A, B). Without RA, no influence of TGF-β or IL5 on the expression of such molecules was observed (Figure 5A, B). However, addition of IL5 to RA with or without TGF-β appeared to enhance the expression of CCR9 on the treated B cell surface (Figure 5A, B). Notably, considerable percentages of B cells expressed either α4β7 or CCR9 on their surface though double expressers were found to be present as well (Figure 5A, B). Under these conditions, PEC B1a and B1b cells showed higher percentages of B cells expressing these two molecules in comparison to PEC B2 cells (Figure 5A). Among splenic B cells, B1a and MZ cells were also higher for such markers in comparison to B2 cells (Figure 5B). Thus, expression of these gut homing molecules seemed to be consistent with the switching tendency of B cell subpopulations to IgA as observed above as well as when checked for the expression of surface IgA on PEC and splenic B cell subpopulations (cultured under various stimulatory conditions) by flow cytometry (Figure 2, Figure 3 and Figure 5C, D).

**Figure 6:**
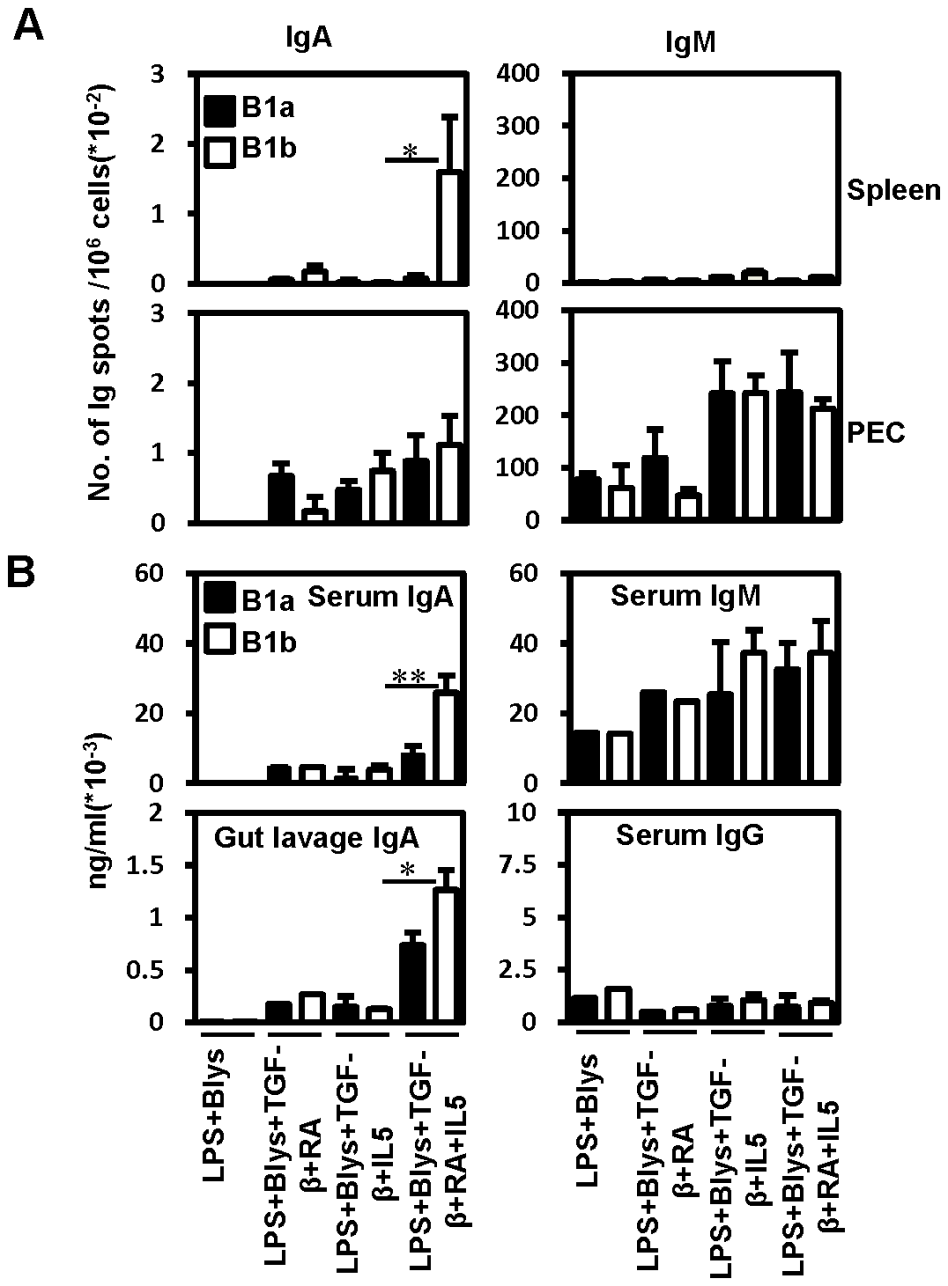
RA and TGF-β treated PEC B1 cells migrate to spleen and gut and produce serum and intestinal secretory IgA. IgA^-^ PEC B1a and B1b cells sorted in a fashion similar to previous experiments were cultured under various IgA CSR inducing conditions for 3 days and transferred intraperitoneally (16000 live cells/mouse) into lymphopenic Rag1^-/-^ recipients. (A) Unstimulated splenocytes and 2 days LPS (25 µg/ml) stimulated PECs of the recipient mice were analyzed for the presence of IgA and IgM producing cells by ELISPOT 2 weeks after adoptive B cell transfer. (B) Levels of secretory Igs in the serum and gut lavage of recipient mice were determined by ELISA. Per group, 2-3 mice were used as recipients. Cells pooled from the recipients belonging to the same group were used for ELISPOT assay and it was done in triplicates. Data show the results of one of two independent experiments. Bars represent mean ± SD.

The expression of gut homing molecules - α4β7 and CCR9 after TGF-β and RA treatment should render these cells capable of migrating to the gut. Therefore, the migratory capacity of such B cells was tested by adoptive transfer experiment. PEC B1a and B1b cells treated *in vitro* with RA and TGF-β for three days were adoptively transferred into lymphopenic Rag1^-/-^ recipients. Day three was chosen because high expression of *Igα* germ line transcripts could be detected after this period of treatment (Figure S2). The recipients were analyzed 2 weeks after transfer for the presence of IgA and IgM ASCs in the spleen, and the peritoneal cavity as well as for the secretory IgA and IgM in the serum and intestinal lavage. Interestingly, presence of IgA SCs could be detected in the recipients of PEC B1 cells that were triggered with TGF-β or RA (Figure 6A and Figure S4A). However, only the splenocytes of recipients of B1b cells treated with TGF-β+RA+IL5 contained considerably high numbers of IgA SCs (Figure 6A and Figure S4A). Higher amounts of secretory IgA could be detected in the serum and gut lavage of the recipients of B1a or B1b cells treated with TGF-β+RA+IL5 in comparison to any other recipient groups (Figure 6B and Figure S4B). Again the recipients of B1b cells were higher in comparison to B1a cell recipients. Flow cytometric analysis of PEC cells from transfer recipients showed the presence and survival of transferred cells (Figure S4C).

Together, these findings demonstrate a synergistic and differential effect of RA and TGF-β on PEC and splenic B cell subpopulations for IgA switching. Surprisingly, B1b cells preferentially switch to IgA independent of T cell help.

## Discussion

Peritoneal cavity B cell subpopulations differ from each other with regard to origin, phenotype and function. PEC B1 cells differentiate faster into plasma cells after stimulation with mitogen than B2 cells from the same compartment.[25], [32] *In vivo*, the two subtypes of B1 cells – B1a and B1b B cells have been shown to react differently to pathogenic infections.[20], [33] In murine infection models, B1a cells have been shown to respond abruptly after infection whereas B1b cells give rise to long lasting memory immunity against certain pathogens.[20], [33] Moreover, these two B1 cell subtypes also have been shown to differ in their ability to produce IgA.[25], [34] Along these lines, recent *in vitro* studies have demonstrated that compared to peritoneal and splenic B2 cells, PEC B1 cells switch more preferentially to IgA in response to a combination of TGF-β, LPS and BLys.[24] However, in this study no attempt was made to distinguish between two B1 cell subtypes. Our previous finding that among PEC B cells, almost exclusively, B1b cells had switched to IgA *in vivo* [25] led us to speculate that B1a and B1b cells might differ in their IgA switching potential under stimulatory culture conditions. When tested, this speculation was found to be true as TGF-β in combination with LPS and BLys led to significantly higher switching of B1b cells to IgA in comparison to the other two PEC B cell subpopulations. Thus, IgA SCs must have resulted from CSR *in vitro* after treatment with TGF-β as the possibility of expansion of preexisting IgA^+^ cells among B1b cells had been already excluded by sorting out IgA^+^ cells before culture.

TGF-β together with RA has recently been shown to induce IgA switching synergistically.[16] In the present study, we used this knowledge to determine the propensity of different peritoneal and splenic B cell subtypes to switch to IgA in response to RA with or without TGF-β. The combined effect of these two factors together with LPS, IL5 and BLys was much stronger than any other combination of stimulants. Again, the frequency of switching to IgA among PEC B cells was significantly higher in B1b cells compared to other PEC B cell types. Interestingly, among splenic B cells, B1a and MZ B cells showed significantly higher IgA CSR than B2 cells. Low switching frequency observed among PEC and splenic B2 cells can be explained by the fact that they require additional stimuli for proliferation and differentiation. For example, B2 cells expressing higher surface IgD show stronger proliferative response in presence of anti-IgD-Dex which is further enhanced by addition of IL4.[23] The considerable high propensity of splenic B1a cell to switch to IgA is consistent with studies where CD5 expressing splenic B1 cells have been claimed to contribute to IgA production.[35]

Besides induction of CSR, the vitamin A metabolite RA is also known to induce or upregulate the expression of gut homing molecules - integrin α4β7 and the chemokine receptor CCR9.[5] Quite consistent with that, in our *in vitro* experimental set up, we observed an upregulated expression of these two gut homing molecules after treatment with RA. Interestingly, the expression of α4β7or CCR9 was higher under conditions that led to enhanced induction of IgA CSR. PEC and splenic B cell subsets prone to enhanced IgA CSR showed higher expression of these homing molecules on their surface. In agreement with the upregulation of gut homing molecules, when PEC B1 cells, treated with a combination of RA, TGF-β, LPS, BLys and IL5, were transferred i.p. into Rag1^-/-^ mice, IgA could be found in serum as well as in gut lavage. This indicates gut migration of *in vitro* switched IgA producing cells.

Isotype switching in B cells is mediated by the enzyme AID.[36] This enzyme is also responsible for nucleotide exchanges leading to somatic hypermutation in the V regions of B cell receptors (BCRs) [36]. Quite expectedly, AID had been found to be expressed in cultured B cells under stimulatory condition. This raised the query whether B cells undergoing IgA CSR also accumulate SHM under our experimental conditions. Interestingly, a considerable fraction of IgA VH sequences derived from RA+TGF-β treated PEC and splenic B1 cells showed nucleotide exchanges. Apparently, like CSR, SHM might also have been induced *in vitro*. Surprisingly, besides IgA VH sequences, IgM VH sequences derived from the same B cell populations also showed higher frequencies of mutations than unstimulated controls.

SHM *in vivo* results in affinity maturation of a particular BCR.[37] This is however accompanied by the generation of many nonfunctional BCRs due to the introduction of nonsense nucleotides by SHM. In accordance, one would expect to find many non-functional VH sequences under *in vitro* conditions. This was apparently not the case. The simplest explanation would be that B cells that had already undergone SHM, and started IgA CSR, are selectively expanded under our culture conditions. Such cells might be qualitatively or quantitatively distinct from B cells that had not yet undergone such processes. Signaling molecules or transcription factors might be up- or down-regulated in this state and the cells might preferentially respond to the stimuli *in vitro*. Alternatively, IgA CSR and SHM were induced *in vitro*. The low frequency of non-functional VH could be explained by an intrinsic selection mechanism to generate functional BCRs. It is known that nonfunctional mRNAs are recognized and such cells might be eliminated.[38] Also B cells producing nonfunctional non-secretable VH or VL chains might be deleted by intrinsic mechanisms. This would imply a strong first round of selection for functional BCRs independent of antigen recognition.

B1 cells are generally believed to react independent of CD4 T cell help.[39], [40] Our findings are quite consistent with this notion. The fact that cytokines together with RA efficiently induced CSR and SHM in such cells without interaction with T cells or the involvement of CD40/CD40L suggests that B1 cells indeed can undergo differentiation without T cell help. This is in contrast with the earlier report where signaling through BCR, CD40 and CD38 were shown to be necessary for the *in vitro* induction of mutations presumably in B2 cells.[41] The factors required for such differentiation are probably provided *in vivo* by stroma or myeloid cells like intestinal DCs or macrophages. B cells themselves could be the source of some of such factors under *in vivo* conditions but also T cells interacting with such B1 cells in a non-cognate fashion might be involved.

In conclusion, our work shows that under stimulatory conditions *in vitro*, PEC B1b cells switch to IgA with a higher propensity in comparison to PEC B1a or B2 cells. The differences among splenic B cell subpopulations with regard to induced switching to IgA are also quite evident. The results of this study show differential stimulatory requirements for different B cell subsets and suggest the presence of distinct mechanisms regulating the induction of IgA CSR among them.

## Supporting Information

Figure S1 Strategy for sorting (A) PEC and (B) splenic B cell subpopulations using FACS. The numbers above the respective gates in the right most panels represent purity of the sorted cells in percentage. Cell sorting was performed using FACSAria® (BD). Dead cells were excluded by applying appropriate gating strategy in the side and forward scatter plot. Doublets from the lymphocyte gated population were excluded by applying appropriate scatter gate. Reanalysis revealed that cells were >95% pure.
(TIFF)

Figure S2 Combined treatment with TGF-β and RA induces the expression of Igα germiline transcript. IgA^-^ PEC and splenic B cells sorted in a fashion similar to previous experiments, were cultured in IgA CSR inducing conditions for two or three days and checked for the expression of Igα germline, and RPS9 (house keeping gene) transcripts by RT-PCR. Results of semi-quantitative PCR, using three serial dilutions (UD – undiluted; 1∶2 and 1∶4) of respective cDNA as template have been displayed. Primers: RPS9, forward (for) 5′- TTGACGCTAGACGAGAAGGAT-3′ reverse (rev) 5′-AATCCAGCTTCATCTTGCCCT -3′; Igα germline transcript, for Iα2 5′- CCAGGCATGGTTGAGATAGAGATAG -3′ rev Cα2 5′-GAGCTGGTGGGAGTGTCAGTG-3′. PCR conditions were: 94°C for 20 s, annealing at various temperatures for 40 s, 72°C for 40 s; 33-38 cycles.
(TIFF)

Figure S3 IgA antibodies derived from TGF-β and RA treated PEC B cells displays reactivity towards gut bacteria. Binding of secretory IgA antibodies present in the culture supernatants of FACS purified IgA^-^ PEC B1a, B1b and B2 cells to gut bacteria was tested by flow cytometry. Sorted cells were cultured with indicated factors for 4 days before collecting the supernatant. After incubation of a mixture of gut bacteria isolated from the normal BALB/c mice (also the source of the PEC cells used for this experiment) with supernatant for 30 minutes, secretory IgA bound to bacteria was revealed by using FITC conjugated anti mouse IgA (BD) antibody. For isolating the gut bacteria, colonic content of the mouse was collected after flushing with PBS, effectively mixed by vortexing and centrifuged at 30 g for 30 minutes to remove the fecal material. Supernatant containing gut bacteria was collected and used for the experiment. Bacteria without addition of antibody (blank) or supernatant or added with isotype-matched control antibody was used as staining controls.
(TIFF)

Figure S4 Survival of cultured PEC B1 cells after *in vivo* transfer into Rag1^-/-^ recipients. Sorted IgA^-^ PEC B1a and B1b cells were cultured under various IgA CSR inducing conditions for 3 days and transferred intraperitoneally (12000 live cells/mouse) into lymphopenic Rag1^-/-^ recipients. (A) Unstimulated splenocytes and 2 days LPS (25 µg/ml) stimulated PECs of the recipient mice were analyzed for the presence of IgA producing cells by ELISPOT 2 weeks after adoptive B cell transfer. (B) Levels of secretory Igs in the serum and gut lavage of recipient mice were determined by ELISA. (C) Percentage (gated for live lymphocytes) and numbers of CD19^+^ B cells in the peritoneum of recipient mice were determined by flow cytometry. PEC cells from individual recipient mouse were analyzed by flow cytometry. Per group, 3–4 mice were used as recipients. Cells pooled from the recipients belonging to the same group were used for ELISPOT assay and it was done in triplicates. Bars represent mean ± SD.
(TIF)
