# Supplementary figures and images for: An Intrinsic Propensity of Murine Peritoneal B1b Cells to Switch to IgA in Presence of TGF-β and Retinoic Acid
Source: PLoS One. 2013 Dec 6;8(12):e82121. doi: 10.1371/journal.pone.0082121 (PMC3855760; doi:10.1371/journal.pone.0082121)

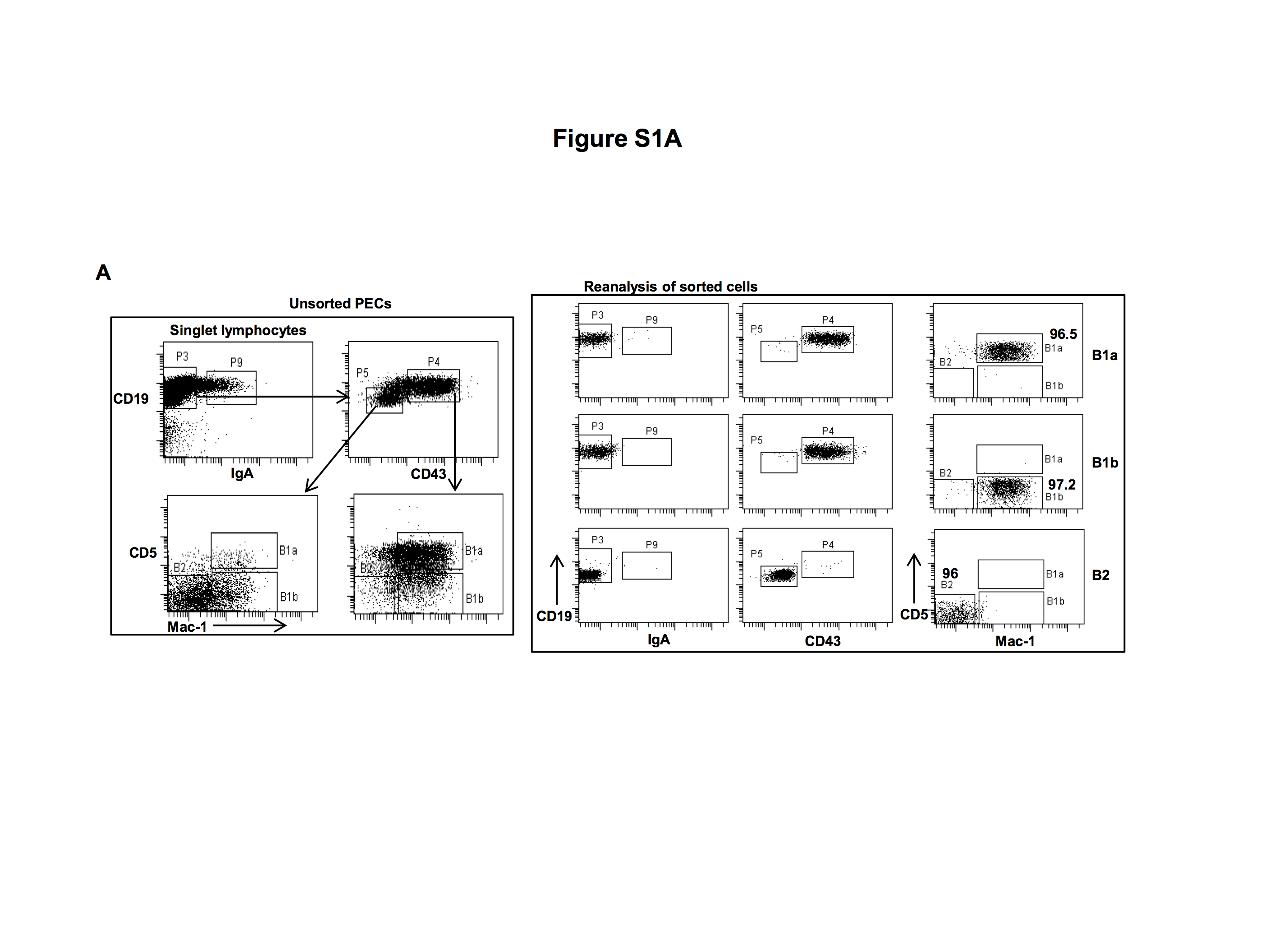

Supplement: Figure S1 — Strategy for sorting (A) PEC and (B) splenic B cell subpopulations using FACS. The numbers above the respective gates in the right most panels represent purity of the sorted cells in percentage. Cell sorting was performed using FACSAria® (BD). Dead cells were excluded by applying appropriate gating strategy in the side and forward scatter plot. Doublets from the lymphocyte gated population were excluded by applying appropriate scatter gate. Reanalysis revealed that cells were >95% pure. (TIFF) [file pone.0082121.s001.tiff]

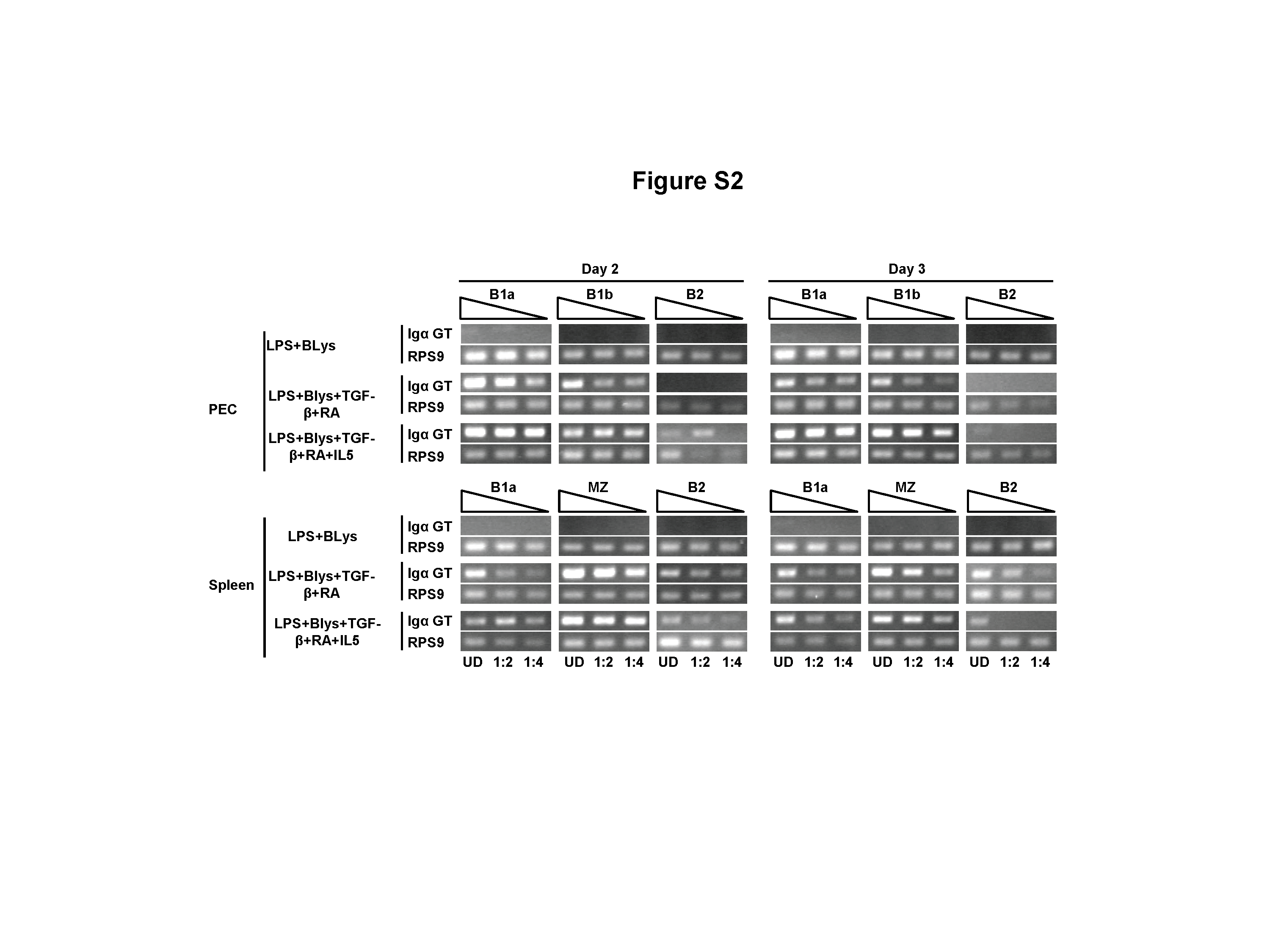

Supplement: Figure S2 — Combined treatment with TGF-β and RA induces the expression of Igα germiline transcript. IgA- PEC and splenic B cells sorted in a fashion similar to previous experiments, were cultured in IgA CSR inducing conditions for two or three days and checked for the expression of Igα germline, and RPS9 (house keeping gene) transcripts by RT-PCR. Results of semi-quantitative PCR, using three serial dilutions (UD – undiluted; 1∶2 and 1∶4) of respective cDNA as template have been displayed. Primers: RPS9, forward (for) 5′- TTGACGCTAGACGAGAAGGAT-3′ reverse (rev) 5′-AATCCAGCTTCATCTTGCCCT -3′; Igα germline transcript, for Iα2 5′- CCAGGCATGGTTGAGATAGAGATAG -3′ rev Cα2 5′-GAGCTGGTGGGAGTGTCAGTG-3′. PCR conditions were: 94°C for 20 s, annealing at various temperatures for 40 s, 72°C for 40 s; 33-38 cycles. (TIFF) [file pone.0082121.s002.tiff]

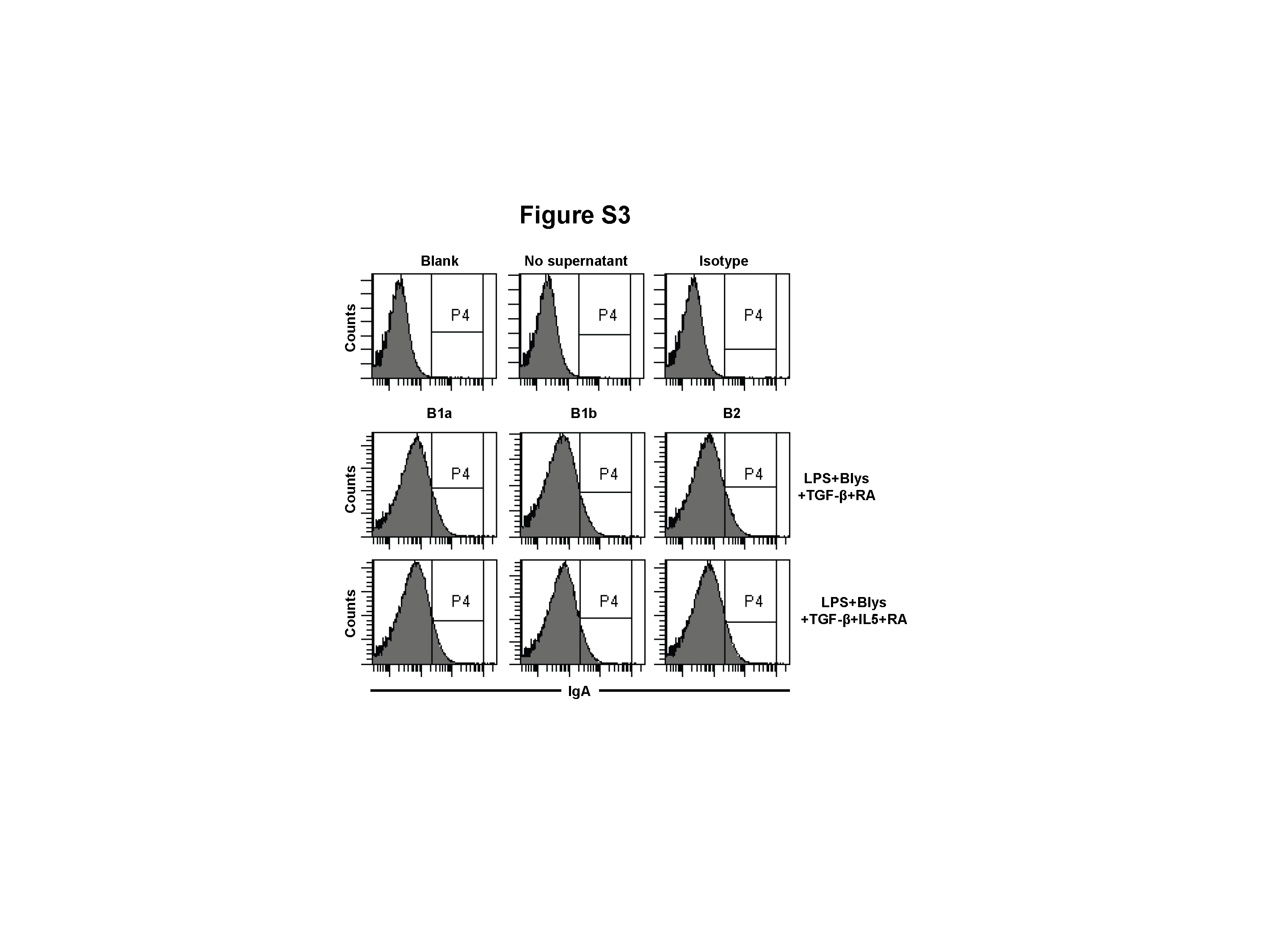

Supplement: Figure S3 — IgA antibodies derived from TGF-β and RA treated PEC B cells displays reactivity towards gut bacteria. Binding of secretory IgA antibodies present in the culture supernatants of FACS purified IgA- PEC B1a, B1b and B2 cells to gut bacteria was tested by flow cytometry. Sorted cells were cultured with indicated factors for 4 days before collecting the supernatant. After incubation of a mixture of gut bacteria isolated from the normal BALB/c mice (also the source of the PEC cells used for this experiment) with supernatant for 30 minutes, secretory IgA bound to bacteria was revealed by using FITC conjugated anti mouse IgA (BD) antibody. For isolating the gut bacteria, colonic content of the mouse was collected after flushing with PBS, effectively mixed by vortexing and centrifuged at 30 g for 30 minutes to remove the fecal material. Supernatant containing gut bacteria was collected and used for the experiment. Bacteria without addition of antibody (blank) or supernatant or added with isotype-matched control antibody was used as staining controls. (TIFF) [file pone.0082121.s003.tiff]
